# Visuomotor perturbation in a continuous circle tracing task: novel approach for quantifying motor adaptation

**DOI:** 10.1038/s41598-019-55241-4

**Published:** 2019-12-10

**Authors:** Erez James Cohen, Kunlin Wei, Diego Minciacchi

**Affiliations:** 1 Department of Experimental and Clinical Medicine, Physiological Sciences Section, University of Florence, Florence, Italy; 2 School of Psychological and Cognitive Sciences and Beijing Key Laboratory of Behavior and Mental Health, Peking University, Beijing, China

**Keywords:** Learning and memory, Motor control, Sensorimotor processing

## Abstract

The study of motor adaptation certainly has advanced greatly through the years and helped to shed light on the mechanisms of motor learning. Most paradigms used to study adaptation employ a discrete approach, where people adapt in successive attempts. Continuous tasks on the other hand, while known to possess different characteristics than discrete ones, have received little attention regarding the study motor adaptation. In this paper, we test for adaptation using a continuous circle tracing task with a visuomotor gain perturbation. To examine the feasibility of this task, 45 normal subjects divided into 3 groups were tested for adaptation, aftereffects, and generalization. All subjects exhibited a gradual adaptation when faced with a perturbation as well as opposite aftereffects once the perturbation was removed. Aftereffects tended to persist unless veridical feedback was given. The task generalized well both in size and in space. We believe that this task, by being continuous, could allow for a thorough investigation of visuomotor adaptation to gain perturbations in particular, and perhaps be expanded to other types of adaptations as well, especially when used alongside discrete tasks.

## Introduction

Motor adaptation refers to the process by which behavior is modified to accommodate a certain perturbation encountered. It is thought to occur not by a simple error correction mechanism, but by a modification of internal representations based on predictions of the eventual outcome of the movement itself^1^. This adaptive process requires the brain to make several assessments regarding the perturbation it is facing. The first of which is whether the perturbation is systematic or transient^2^. The distinction between the two will determine whether a change in behavior is necessary, as transient perturbations should not elicit an adaptive response and would, therefore, be solely compensated. Systematic perturbations, on the other hand, would merit a consequent change in behavior, as the perturbation cannot be attributed to randomness^3^. This would result in a gradual improvement following the abrupt presentation of the perturbation. If adaptation has occurred, then once the perturbation is removed, reverting to normal performance would occur at a certain delay, what is commonly known as “aftereffects”^4^, resulting in errors opposite to those present initially when the perturbation was introduced. Furthermore, if indeed an internal representation was modified, the adapted behavior would be expected to generalize also to other circumstances^5,6^.

The study of motor adaptation commonly employs the use of discrete tasks (e.g., reaching), operating on a trial-to-trial basis, evaluating the trial-by-trial learning or cumulative learning over successive trials. This approach is inherently long and may require hundreds of trials (e.g.^7^), although, it was suggested that even a few trials may produce long-term retention in some cases^8,9^. Still, when using a trial-by-trial approach, the presence of an inter-trial interval is inevitable. Though this interval was shown to be an important factor for learning, as it may affect the decay of learning, the extent by which it may affect learning is not well characterized^10,11^. Still, the mere presence of an inter-trial interval, by leaving room for preparation time which could influence the extent of learning^12^, may represent a confounding factor when interpreting results and, therefore, must be meticulously controlled if we wish to compare subjects.

In addition, a trial-by-trial assessment is accompanied by several uncertainties regarding the performance. For example, when moving to different directions, as is often the case in reaching adaptation paradigms e.g.^13–17^, each trial is accompanied by an initial uncertainty regarding the location of the target. A second uncertainty is related to the nature of the perturbation, whether it is persistent (i.e., always present) and consistent (i.e., always of the same magnitude). The third is whether the perturbation is specific to a single direction, or present in all directions equally. Although subjects will eventually adapt also in noisy environments and conditions, these uncertainties are likely to generate compensatory responses that are not a manifest of adaptation per se. This is evidenced in force field perturbation paradigms where people use impedance control against uncertain perturbation^18–20^. While impedance does reduce as the internal model is learned and updated^21^, it could potentially contaminate the results when the adaptation period is limited. Though it remains debatable whether and to what extent impedance control plays a role in other adaptation paradigms (e.g., rotation perturbation), uncertainty surely plays an important role in motor adaptation and all of these factors may affect both performance as well as the interpretation of data.

It is possible to eliminate some of these uncertainties. For example, testing for a single direction may eliminate the uncertainty regarding the target’s location as well as the perturbation specificity. However, which direction is better? direction specificity was shown to play an important role in adaptation^22,23^ and, as such, the elimination of different directions may produce incomplete results, especially when considering that baseline performance is already direction specific. On the other hand, testing more directions will reduce use-dependent learning^24^ and increase the number of trials.

It should be noted that these “issues” related to discrete tasks may also be desired, especially when we wish to examine certain specific aspects relative to components of motor adaptation. For example, modulation of the inter-trial interval may be used to favor explicit components of learning e.g.^12^. Moreover, under certain circumstances, we may wish to examine the effects of a certain direction on adaptation and, therefore using different directions may be important^23^. However, if we do wish to conduct a study in which the effects of these factors are reduced, we could opt to use motor adaptation that involves continuous movements e.g.^25–28^. In fact, continuous adaptation does not require preparation time and, as such, should provide more consistent results by removing this confounding factor. Moreover, as the movement is continuous, any uncertainty related to either direction or the perturbation itself is eliminated. Trial-by-trial decay of learning might also be reduced, thus adaptation can potentially be achieved rapidly.

In this paper, we examined motor adaptation to a visuomotor gain perturbation using a continuous task of circle tracing. This specific task was chosen since it was shown to provide consistent results across measurements^29^. Moreover, by being a simple and continuous task it may greatly reduce any confounding factors related to the inter-trial interval as well as, by being a circle, to direction biases and specificity. Even though a circle-drawing task was previously used to assess explicit and implicit motor adjustments^30^, whether the motor adjustment observed in the previous work can be viewed as adaptation is questionable. Specifically, in the previous study a gain perturbation was presented for a single revolution, and immediately tested the aftereffect with veridical feedback in the following 2–3 revolutions. However, since motor adaptation involves a modified forward internal model and typically yields an aftereffect and limited generalization of adaptation across movement contexts, to affirm that adaptation had occurred both aspects should be examined. In the previous study, the former aspect was not shown, seeing that veridical feedback was immediately provided, and the latter aspect was not examined. Therefore, whether or not visuomotor gain perturbations in a continuous circle-drawing task can lead to successful adaptation has not been rigorously examined. To overcome these issues, we have examined adaptation for a large perturbation and over a longer period, as well as two types of generalizations.

## Materials and Methods

### Participants

45 healthy adults were recruited for this study (age: 20.35 ± 2.98 years; 21 males). All participants were right handed. Participants were naive to the task and the purpose of the study and free of documented neurological impairments. All participants reported having a corrected-to-normal visual acuity. The study protocol was approved by the Institutional Review Board of Peking University and all procedures conformed to the code of ethics of the Declaration of Helsinki. All participants gave written informed consent and were paid for their time.

### Set up and task

**Figure 1:**
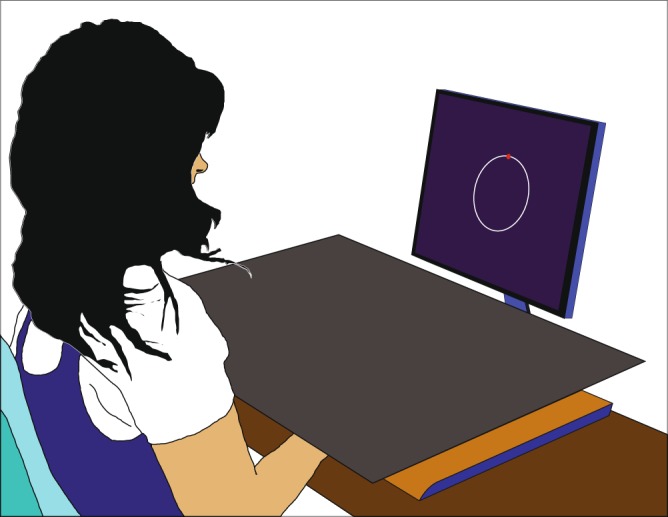
Setup. Diagram illustrating the experimental setup. Each subject was presented a circle template projected on a monitor in front of her/him at eye level. A black paperboard occluded vision of the hand. The subjects executed tracings of a circle, while seated without the support of either wrist, arm, or elbow, in such a way that the only contact with the tablet was made through the pen.

Participants were presented a circle template projected on a monitor mounted vertically in front of them at eye level (Fig. 1). A black paperboard occluded vision of the hand. On the circle template (5.4 cm in radius) a small red moveable circle represented the starting point. The participants were instructed to execute tracings of a circle, using graphic pen tablet (Wacom Intuos® PTK-1240, Tokyo, Japan; active area: 462 × 305 mm), while seated without the support of either wrist, arm, or elbow, in such a way that the only contact with the tablet was made through the pen. Further instructions included tracing the target circle counterclockwise as fast as they can while still being accurate. Before starting the task, each participant was asked whether the instructions were understood. Once participants positioned themselves at the correct point, the small circle turned to green indicating the start of the trial and the cursor became invisible. During execution, the cursor position, represented by the small circle, was visible. The cursor trajectory was also visible and was reset every revolution. The position of the small circle and the cursor trajectory will be referred to as cursor feedback. Each participant was tested individually.

### Experimental design

**Figure 2:**
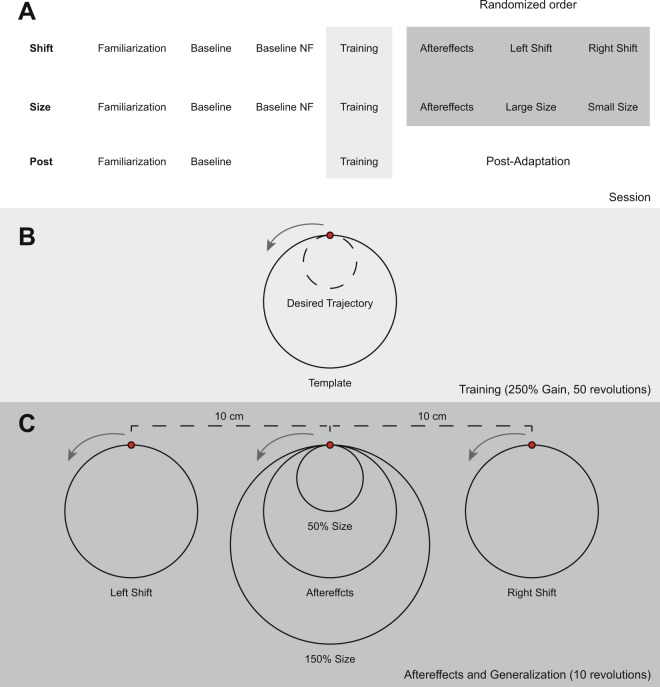
Experimental design. (**A**) Schematics of the session organization. Subjects were divided into one of three groups: Shift, Size and Post. For both Shift and Size groups, the session consisted of the following trials: familiarization (10 revolutions with veridical feedback), baseline (10 revolutions with veridical feedback), no feedback baseline (Baseline NF; 10 revolutions with no feedback), training (50 revolutions with 250% gain perturbation to cursor feedback), aftereffects and two generalization trials (left and right shift for Shift group, large and small size for Size group, each consisting of 10 revolutions with no feedback). The order of the last 3 trials (i.e., aftereffects and generalization trials) was randomized. For the Post group, the session consisted of the following trials: familiarization, baseline, training, and post-adaptation (10 revolutions with veridical feedback). (**B**) A diagram demonstrating the circle template presented to the subjects. During the trial, the cursor position was represented by the small circle (shown in red). Subjects were asked to trace the circle according to the template. During the baseline trial, the desired trajectory corresponded to the circle template. During training, a 250% gain perturbation to the cursor feedback was introduced. In order to match the template subjects needed to draw a circle 40% smaller than the original (i.e., desired trajectory; represented by the dashed line). (**C**) Diagram showing the different templates used for the aftereffects and generalization trials. In these trials no feedback was presented. For the aftereffects, the circle’s template presented was the same size as the original, located at the same place (i.e., Aftereffects). For Shift group, the circle’s template was of the same size, shifted to the left (i.e., Left Shift) and to the right (i.e., Right Shift). For Size group, the circle’s template was 50% the size of the original (i.e., 50% Size) and 150% the size of the original (i.e., 150% Size) presented at the same place as the original.

Subjects were divided into three groups (n = 15/group; 7 males/group), two generalization groups (size and spatial generalization; i.e., Size and Shift), and a post-adaptation group (i.e., Post; Fig. 2). Subjects were trained to adapt to a gain perturbation of the cursor position about the origin of the circle. To achieve a robust effect, perturbation was set to 250% gain, causing the desired tracing of the circle to be 40% of the original circle size (40% of 5.4 cm i.e., 2.16 cm in radius). In the generalization group each session consisted of the following trials: familiarization (10 revolutions of a circle with veridical cursor feedback), baseline (10 revolutions of a circle with veridical cursor feedback), baseline no feedback (i.e., Baseline NF; 10 revolutions of a circle with no cursor feedback), training (50 revolutions of a circle with a gain perturbation of 250% of the cursor), aftereffects (10 revolutions of a circle with no cursor feedback), and generalization (2 trials each consisting of 10 revolutions with no cursor feedback).

For the Shift group, the target circle’s template was shifted 10 cm to the left or to the right of the original circle, one trial for each. For the Size group, the circle’s template presented was either bigger (150% of the original radius, i.e., 8.14 cm) or smaller (50% of the original radius, i.e., 2.71 cm), one trial for each. For Size and Shift groups each subject performed both generalization trials related to the group (either large and small size or left and right shift, respectively), along with aftereffects trial. For these groups, the order of the aftereffects and generalization trials was randomized.

In the Post group, each subject participated in one session consisting of baseline (10 revolutions with veridical cursor feedback), training (50 revolutions with gain perturbation of 250% of the cursor) and a post-adaptation trial (10 revolutions of a circle with veridical cursor feedback).

### Analysis

**Figure 3:**
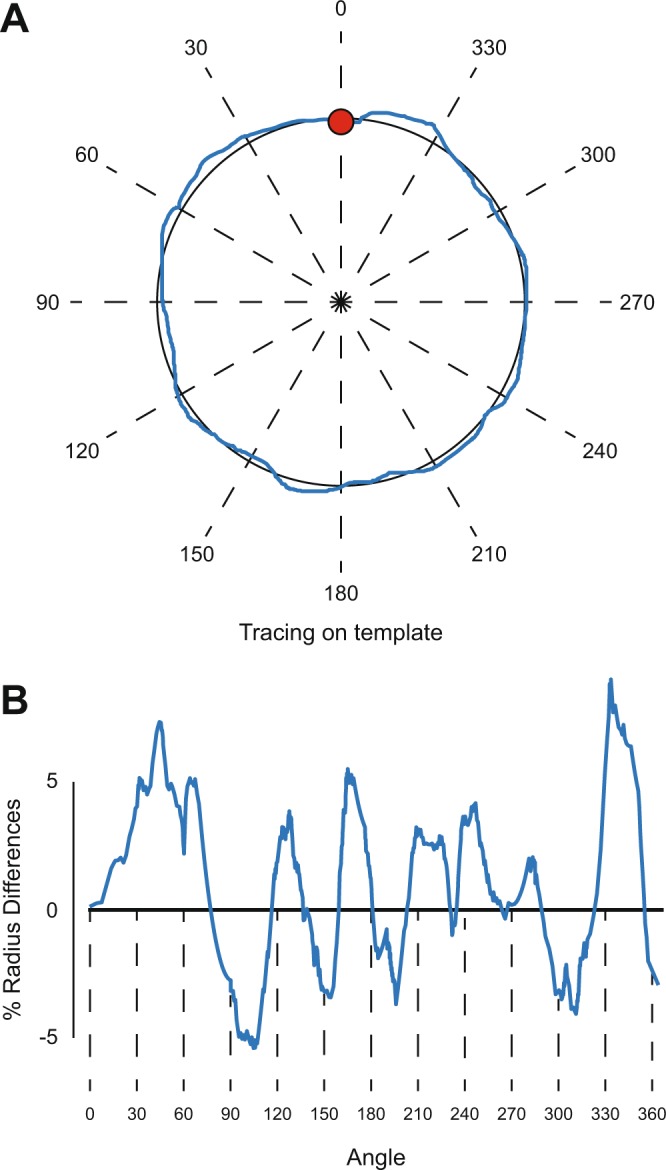
Circles analysis. An example of the calculation of the precision of a traced circle. (**A**) A complete revolution of a traced circle drawn counterclockwise (blue line) on a template is shown. The center of the template was used to measure the radius of each point on the tracing. (**B**) For each angle of the circle, the template’s radius was subtracted from the measured radius at that specific angle in order to obtain the Radius Difference, which was divided by the template’s radius to obtain % Radius Difference for every direction of the circle over time.

Following data collection, circle tracing analysis consisted of calculation of traced circle radii, measured as point distances from the template’s center (i.e., radius). For each measured radius, deviations from the template’s radius (i.e., reference radius) were also calculated (i.e., residual difference; RD) and are presented as percent deviation from the expected radius (i.e., % radius difference; %RadD; Fig. 3). Point direction was calculated as the angle difference from the starting position, considering the circle center as the vertex.

The amount of learning was derived from %RadD of the expected adapted radius and is presented as %Learning, where 100% represents a complete adaptation (i.e., %RadD equals zero for the expected adapted radius). Learning rate was estimated as the %Learning averaged over the first 3 revolutions of the training trial. Unless stated otherwise, all %RadD and %Learning values are presented as group mean and standard deviations.

### Statistics

To evaluate any potential between-group difference a One-Way ANOVA was implemented on the average %RadD of the baseline performance of each subject. To evaluate any differences that may relate to adaptation between groups a One-Way ANOVA was also implemented on the %Learning during training for each subject.

To evaluate the immediate extent of learning, a two-tailed t-test of %Learning was conducted comparing the mean of last 3 revolutions during training with the first evolution of the following trials (i.e., aftereffects, generalization, post-adaptation). As a measure of the significance of decay, the average of the first 3 revolutions of each trial was compared with the average last 3 revolutions of said trial using a two-tailed t-test. To evaluate potential within-trial decay in the generalization groups, the average %Learning of the last 3 revolutions of the aftereffects and generalization trials was also calculated and compared with the average of the first 3 revolutions using a two-tailed t-test.

## Results

The adaptation and subsequent tests of aftereffect and generalization were performed in a couple of minutes. Across all subjects and trials, the average completion time for the entire session was 126.32 ± 35.7 seconds.

First, we found no significant differences in baseline performance between groups (F_2,42_ = 0.88, p = 0.42, one-way ANOVA). Baseline precision (measured as %RadD) across subjects averaged −1.81 ± 3.87%. For every subject, baseline performance was shown to be relatively consistent with small variations, estimated for each subject as the standard deviation of the %RadD computed across all revolutions and then averaged, overall averaging at 4.72 ± 1.69%.

During the training trial, all subjects, in all groups, showed an immediate increase in error upon the introduction of visuomotor gain adaptation, averaging 38.1 ± 35.5%Learning for the first revolution. Then, they exhibited a gradual reduction in error, with an average learning rate of 63.4 ± 21.7% by the end of the third revolution. Also in this case no significant differences were found between groups (F_2,42_ = 0.27, p = 0.76, one-way ANOVA). As a measure of the extent of learning during training, the average of the last 3 revolutions was calculated for each group and measured 95.5 ± 8.1% for the Size group, 93.5 ± 12.2% for the Shift group and 93.7 ± 4.5% for the Post group. At the end of the session, all our subjects reported to be aware of the perturbation.

**Figure 4:**
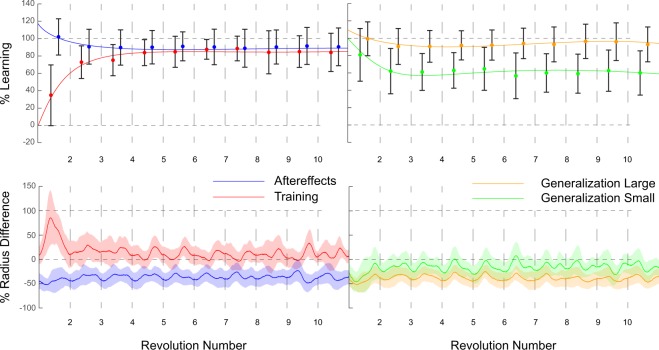
Results Size group. Results obtained for the Size group (n = 15) presented as %Learning (upper panel) and % Radius difference (lower panel). On the left side are the results obtained from both training (red; only first 10 revolutions) and aftereffects (blue), whereas on right side are the results obtained from the large generalization (orange) and small generalization (green). For %Learning, the dots represent the revolution’s mean whereas the error bars represent the standard deviation for said revolution; the solid lines illustrate the general trend during the trials. In the lower panel % Radius difference are presented as mean (line) and standard deviation (shaded areas). It is possible to note that subjects maintain their adapted state throughout with the exception of the small generalization, in which values demonstrated a slight reduction in adaptation.

**Figure 5:**
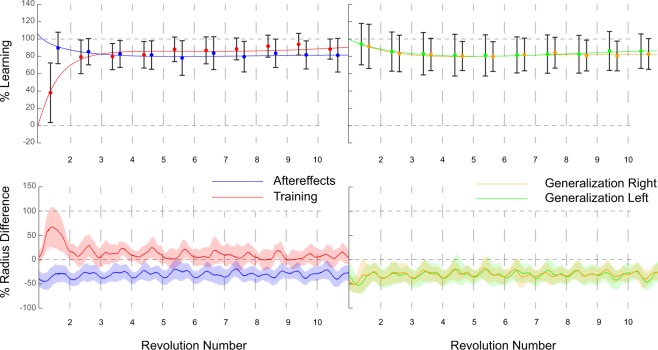
Results Shift group. Results obtained for the Shift group (n = 15) presented as %Learning (upper panel) and % Radius difference (lower panel). On the left side are the results obtained from both training (red; only first 10 revolutions) and aftereffects (blue), whereas on right side are the results obtained from the right generalization (orange) and left generalization (green). For %Learning, the dots represent the revolution’s mean whereas the error bars represent the standard deviation for said revolution; the solid lines illustrate the general trend during the trials. In the lower panel % Radius difference are presented as mean (line) and standard deviation (shaded areas). It is possible to note that subjects maintain their adapted state throughout all trials with a very small decay.

**Figure 6:**
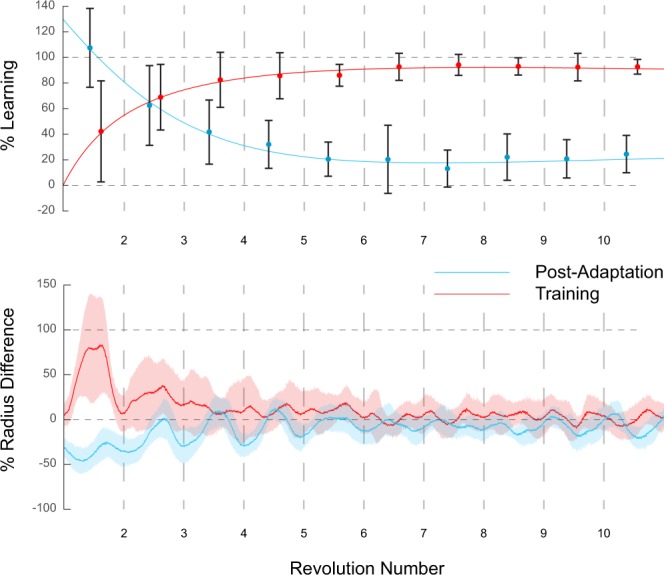
Results Post-adaptation group. Reported are the results obtained for the Post group (n = 15). Results for training (red) and post-adaptation (blue) are shown reported as mean (dots) and standard deviations (error bars) of %Learning (upper panel) and mean (lines) and standard deviations (shaded areas) of % Radius difference of the expected radius (for training is 40% of the original). As expected, the two trials present opposite trends with training initially having larger radii gradually reducing and post-adaptation having smaller radii gradually increasing.

The immediate extent of learning following training for the Size group averaged 101.7 ± 20.8% for the aftereffects trial, 80.6 ± 30.3% for the small size, and 99 ± 19.4% for the large size (Fig. 4), all of which were not found to be significantly different compared to the average of the last 3 revolutions during training (p = 0.34, p = 0.1, and p = 0.55). For the Shift group, the extent of learning averaged 89.3 ± 18.1% for the aftereffects trial, 93.7 ± 23.9% for left shift and 91.3 ± 25.6% for right shift (Fig. 5). Also in this case no significant differences were found between the trials and the last 3 revolutions of the training trial (p = 0.34, p = 0.97, and p = 0.74, respectively). For the Post group, immediate extent of learning averaged 107.2 ± 30.8% (%Learning of the first revolution; Fig. 6), not showing any significant differences with the average of last 3 revolutions of training (p = 0.13).

No significant within-trial decay was found for any of the trials of the Generalization groups. Specifically, aftereffects measured as %Learning of the adaptive state, averaged 85.6 ± 15.4% for the first three revolutions, decaying to 81.1 ± 16.8% (p = 0.1) for Shift, and 93.9 ± 18.9%, decaying to 90.5 ± 20.3% (p = 0.1) for Size. For Shift group, %Learning averaged 87.1 ± 23.2% for left shift, decaying to 84.5 ± 20.8% (p = 0.42); for right shift %Learning averaged 85.4 ± 20.9%, decaying to 81.2 ± 17.1% (p = 0.1). For Size group, small size averaged 67.8 ± 24.4%, decaying to 60.4 ± 23% by the last 3 revolutions (p = 0.13). For large size %Learning averaged 93.2 ± 17.9% and presented no decay, reaching 94.8 ± 20.3% (p = 0.6) by the last 3 revolutions. In sum, generalization across sizes and space was large with small decay over time.

To examine decay across trials for the generalization groups, the average of each trial according to its order was calculated as %Learning of the last 3 revolutions. For the first trial after training, %Learning averaged 91.5 ± 18.7%, for the second 84.6 ± 20.4% and for the third 80.5 ± 24.5%. Revealing an inter-trial decay of learning of ~5.5%.

For the Post group, as expected, subjects presented opposite trends during training and post-adaptation as shown by the change in %RadD. For training, initial %RadD for the first 3 revolutions averaged 30.9 ± 25.2% than expected radius (64.2 ± 26.4%Learning). For post-adaptation, subjects initially performed smaller radii, averaging −21.1 ± 8.6% (70.2 ± 24.6%Learning) by the third revolution. These trials presented converging trends as they continued and by the tenth revolution %RadD values measured 1.85 ± 8.69% for training and −6.8 ± 6.1% for post-adaptation (Fig. 6).

## Discussion

The results in this study appear to satisfy the prerequisites for visuomotor adaptation to gain perturbations. Specifically, we observed a gradual reduction in error after the introduction of the perturbation as well as the presence of opposite aftereffects once the perturbation was removed. Furthermore, the task generalized in space and size, showing a relatively small decay in time. Interestingly, the extent of adaptation appears to be long-lasting when no veridical feedback is presented, as shown by both the high retention of adaptation as well as the low decay during generalization.

Using this task, several of the issues related to discrete measurements indeed may be eliminated. Specifically, there is no inter-trial interval and, as such, any issue related to preparation time^12^ or decay of learning is minimized^10,11^. Furthermore, since the perturbation in this case is evident and consistent, the uncertainties related to the nature of the perturbation are greatly reduced. Also, the use of a circle, by covering all possible directions, reduces direction-related issues. It should be considered, however, that depending on the nature of the study, the characteristics of the discrete measurements may indeed be desired in order to investigate certain aspects related to motor adaptation. Therefore, the implementation of a continuous approach could not substitute a discrete one, and the two are best implemented alongside.

The continuous approach holds an additional advantage compared to other paradigms. In reaching paradigms, it is important to consider the possibility of inaccuracies between measurements, both within a single study as well as between labs. A simple example from reaching adaptation paradigms is whether the reach angle is calculated at end point or at peak velocity. Seeing that this discrepancy is known, some studies integrate these two measurements e.g.^31^. This would result in an operational tool for assessment, but not a precise one. Similarly, discrepancies may also derive from how the start position of a reaching movement is defined (i.e., subjects’ actual start position or an imposed start point). If 4 mm around an imposed starting position is defined as the area of starting position e.g.^23^, the maximum deviation of actual start position from the imposed starting position alone can generate a 6.5° direction deviation between measurements with a reaching distance of 70 mm. As there is no consensus regarding the area around the center for the starting point, these deviations could be even greater e.g.^17^. All of these minor issues could confound the learning data and its interpretations. These discrepancies between measurements could also occur for continuous tasks, such as circle drawing, in which circle metrics may be calculated in different ways e.g.^29,32^. In the circle tracing paradigm, since the measurement for adaptation is continuous, these possible inaccuracies are eliminated, as the starting point is constant and the target is fixed and uniform across all measurements.

A general limitation of this study is the use of a single type of perturbation, i.e., a visuomotor gain perturbation. It should be noted that not all perturbations are alike. Specifically, gain perturbations are adapted quicker and generalize better than visuomotor rotation perturbation^9^. As such, the results presented in this study cannot be extended also to other types of perturbations (e.g., visuomotor rotation). However, we believe that future studies would indeed benefit from the evaluation of a visuomotor rotation perturbation using a continuous task as suggested in this study. Furthermore, it should be noted that a continuous task also presents limitations compared to a discrete one (e.g., directional generalization cannot be tested using a continuous task). Therefore, considering the differences between discrete and continuous tasks, it would be interesting to examine motor adaptation using a combination of both continuous and discrete tasks. This could permit a more thorough investigation of transfer from one modality to the other, as well as to better characterize the differences between the two. Finally, it should be noted that, granted the size of the perturbation used in this study, the observed adaptation may be due to strategic learning. Under this view, it is possible that subjects would demonstrate strategic adjustments, sensitive to goal-based performance error, during the experiment rather than adaptation proper, sensitive to prediction errors between desired and actual consequences of planned movement^33^. We should consider, however, that the line between gradual adaptation and strategy formation is not easy to define. Explicit report of strategy in the current paradigm (if there is any) will be hard to put into numbers. If we assume the Post group explicitly knew about the gain change when they suddenly received veridical feedback after adaptation, they can pull off their strategy during this washout (i.e., post-adaptation). In this case, if an explicit strategy can fully account for the reported adaptation, we should observe an abrupt de-adaptation. However, this is not what we observed. Even though we indeed saw this group de-adapted faster than other groups, their de-adaptation was still gradual. Thus, we postulate that learning performance here contains implicit learning, though the extent of which is not easily quantifiable. Just like rotation adaptation, the relative size of explicit and implicit components might be a function of perturbation size (gain size here). However, this question shall be left for future studies.
